# Young people’s romantic relationships and sexual activity before and during the COVID-19 pandemic

**DOI:** 10.1186/s12889-021-11818-1

**Published:** 2021-10-02

**Authors:** Jennifer Yarger, Abigail Gutmann-Gonzalez, Sarah Han, Natasha Borgen, Martha J. Decker

**Affiliations:** 1 Philip R. Lee Institute for Health Policy Studies, University of California, San Francisco, 490 Illinois Street, Floor 7, San Francisco, CA 94158 USA; 2 Bixby Center for Global Reproductive Health, University of California, San Francisco, 1001 Potrero Avenue, UCSF Box 0842, San Francisco, CA 94110 USA; 3 University of California, Berkeley School of Public Health, 2121 Berkeley Way, Room 5302, Berkeley, CA 94720 USA; 4 Department of Epidemiology and Biostatistics, University of California, San Francisco, 550 16th Street, Floor 2, San Francisco, CA 94158 USA

**Keywords:** Adolescent, COVID-19, Social distancing, Sexual activity, Young adult, Romantic relationships

## Abstract

**Background:**

Social distancing measures to reduce the spread of COVID-19 may profoundly impact young people’s relationships. This study compared adolescent and young adults’ romantic relationships and sexual activity before and after social distancing policies were enacted.

**Methods:**

In June 2020, 351 youth participating in an ongoing intervention study in Fresno County, California completed an online survey about their experiences related to COVID-19. The survey included open and closed-ended questions about their romantic relationships, sexual activity, and online romantic or sexual interactions before and during social distancing restrictions. We used the chi-square test of independence to compare adolescent (ages 13–17) and young adults’ (ages 18–21) responses. Results were also compared to responses in the intervention study’s baseline survey.

**Results:**

One-third (37%) of youth were dating or in a romantic relationship and 28% spent time in person with a partner early in the COVID-19 pandemic. Among those dating or in a relationship, 34% physically distanced from their partner due to parental restrictions related to COVID-19. Youth also spent less time in person with their partners during the pandemic than before. Although most youth (69%) were not sexually active before or during the pandemic, 22% had sex during the social distancing period. Young adults were more likely to spend time with their partners and have sex during the restrictions than adolescents. Most youth were not involved in sexting or online dating, before or during the pandemic.

**Conclusions:**

Adolescents and young adults have continued to engage in sexual and romantic relationships during the COVID-19 pandemic, although many reported physical distancing from their partners. Results suggest that youth continue to need access to sexual health education and services during emergencies such as the COVID-19 pandemic.

## Background

In the early months of the COVID-19 pandemic, state and local governments throughout the United States recommended minimizing contact between people, leading to the closure of schools and nonessential businesses, and shelter-in-place orders to reduce the spread of the disease. In some states, including California, these restrictions continued, particularly school closures. These social distancing measures have profoundly impacted the social lives of adolescents and young adults.

Social distancing from friends and romantic partners may be particularly challenging during adolescence, which is a period marked by a greater need for social connection and peer acceptance and increased risk taking [1, 2]. Popular and news media have portrayed young people as careless and unconcerned about becoming infected with COVID-19 [3, 4]. However, a growing body of research has found concern about COVID-19, support for social distancing guidelines, and participation in social distancing among adolescents and young adults [5–7].

Very little is known about how the pandemic has affected young people’s romantic and sexual relationships, which can have potential implications for positive, as well as negative, developmental outcomes [8, 9]. Researchers have posited that social distancing and stay-at-home guidelines have resulted in less partnered sex for most young people as they face increased parental monitoring and reduced privacy [10]. Studies in adult populations have found evidence of significant declines in sexual activity during the pandemic, although adults did not completely stop engaging in sexual activities [11–14], as well as evidence of increased romantic relationship conflict [15, 16]. Comparatively few studies in this area have included adolescent or young adult populations. In one study of adolescent sexual minority males, participants reported seeing their sexual partners less often during the COVID-19 pandemic [17]. In another study with college students aged 18–25, most students reported a decrease in sexual activity [18]. Just as patterns of relationship involvement and sexual activity vary between adolescence and young adulthood [19, 20], additional research is needed to compare the pandemic’s impact on romantic and sexual relationships by age.

COVID-19 and social distancing guidelines may lead to an increase in young people’s online romantic or sexual interactions. Previous research has found that some adolescents initiate new relationships online [21–23], and young people often communicate with their partners through phone or text, including sexting [24, 25]. During the pandemic, youth may have turned more to forms of digital communication for romantic or sexual interactions, including online dating, sexting, virtual sex and other activities [10]. Research has found an increase in pornography viewing in the pandemic [26], including an increase in pornography use among adolescent sexual minority males [17].

The purpose of this study is to assess the impact of the COVID-19 pandemic on romantic relationships and sexual activity among an understudied population of predominantly Latino young people in Fresno County, California. Specifically, we ask if the pandemic resulted in: 1) physical distancing from romantic partners, 2) less sexual activity, and 3) more romantic or sexual interactions online. We also compared the impact of the pandemic on romantic relationships and sexual activity between adolescents and young adults.

## Methods

### Setting

Fresno County, located in the Central Valley of California, is the most productive agricultural county in the state and nation [27]. Despite this, the county has significantly higher percentages of total (61%) and youth (78%) concentrated poverty compared to the state averages of 30 and 46%, respectively [28]. Over a quarter of Fresno County’s population is aged 18 and younger (28%) and 54% of the population identifies as Hispanic or Latino [29].

On March 19, 2020, California issued the first statewide shelter-in-place order to mitigate the spread of COVID-19, directing all residents to stay home except to go to an essential job or seek basic necessities, such as food, prescriptions, and health care [30]. At the end of May 2020, California had approved Fresno County to begin reopening some sectors, including curbside retail, manufacturing, offices, childcare, and restaurants [31]. Businesses wishing to reopen were required to implement safety precautions such as masking indoors, limiting capacity, and screening employees daily for respiratory illness. However, rising COVID-19 cases and hospitalizations led to a statewide mandate for all Californians to wear masks in public on June 18, 2020, and to close most businesses again in mid-July, 2020 [32]. Virtually all of the schools in Fresno remained closed to in-person instruction throughout the majority of the 2020–21 academic year, offering distance learning instead. Compared to the rest of California, Fresno and other Central Valley counties have been disproportionately impacted by COVID-19. Throughout the summer of 2020, Fresno County had significantly higher rates of COVID-19 compared to California, and the test positivity rate as of February 2021 remained higher than the state average [33].

### Participants and procedures

Participants were selected from a sample of youth enrolled in a five-year, cluster randomized controlled trial of a sexual health education intervention in Fresno County, California [34]. Participants were initially recruited from 49 different sites, including alternative schools, foster homes, and afterschool programs. Youth were initially eligible to participate in the larger study if they lived in Fresno County, were aged 13–19, and spoke English or Spanish. The original study procedures included completing online surveys at baseline and at 3 and 9 months after baseline. Participants received a $10–$20 gift certificate after completing each survey. Passive parental or guardian consent, meaning parents or guardians needed to sign and return the consent form for refusal, was used per state guidelines for all sexual health education offered to youth [35]. Participants completed a consent/assent form at initial recruitment and prior to each subsequent survey.

In June 2020, 797 participants (out of 1260 who enrolled in the larger study) were invited to complete a supplemental online survey. We did not send the supplemental survey to 463 participants who were due to complete the 3-month or 9-month follow-up survey for the larger study. Non-respondents received reminder emails and/or text messages, and study researchers called a subset of participants (*n* = 30) who had started the survey to encourage them to complete it. Before starting the online survey, participants reviewed information about the study and checked a box confirming informed consent. The survey included questions on how COVID-19 had changed participants’ living arrangements and employment, use of technology, education, relationships, sexual and reproductive health, and health service use. The median time to complete the survey was 13 min. All participants received a $10 electronic gift card after completing the survey. This study was approved by the Institutional Review Board at the University of California, San Francisco.

### Measures

All of the measures used in the analyses were collected in the supplemental survey, except where noted below.

#### Physical distancing from intimate partners

Participants were asked if they had “dated or been in a romantic or sexual relationship with anyone” in the last 3 months. If so, they were asked if they “spent time in person with someone you dated or were in a romantic or sexual relationship with” in the last 3 months and if they began living with someone they were dating as a result of the COVID-19 pandemic.

Two items assessed how often they spent time in person with an intimate partner; one item asked, “BEFORE the coronavirus restrictions, how often (on average) did you do the following: Spend time with a date or romantic partner (in person)” and another item asked, “In the last 3 MONTHS (during the coronavirus restrictions), how often (on average) did you do the following: Spend time with a date or romantic partner (in person).” Response options ranged from 1 (never) to 5 (daily).

#### Reasons for physical distancing from intimate partners

If participants reported an intimate relationship in the past 3 months, they were asked, “In the last 3 MONTHS, did you NOT spend time in person with someone you were dating or in a romantic or sexual relationship with for any of the following reasons? Check ALL that apply.” The response options are shown in Table 3. Participants also were asked to rate their agreement with the statement: “If you’re only dating one person, it’s okay to see each other during shelter-in-place.” Response options ranged from 1 (strongly agree) to 4 (strongly disagree).

#### Sexual activity

In the baseline and supplemental surveys, participants reported if they had ever had oral, vaginal, or anal sex and if they had had oral, vaginal, or anal sex in the past 3 months. Two items assessed how often participants had sex before the COVID-19 restrictions and in the last 3 months. The response options ranged from 1 (never) to 5 (daily).

#### Romantic or sexual interactions online

Participants were asked, “As a result of the coronavirus and shelter-in-place restrictions, how has the amount of time you spend doing the following changed,” followed by a list of online activities. The activities included “sexting (sending sexually explicit photos or messages),” “online dating/hook up sites (such as Bumble, Tinder, Hot or Not),” and “watching porn.” Response options were more time, about the same, less time, and I do not do this/does not apply.

#### Open-ended question

Participants were asked the open-ended question, “How has COVID-19 affected your dating or romantic or sexual relationships?”

#### Age group

We created a binary variable for developmental age group at the time of the supplemental survey. Adolescents were defined as ages 13–17 and young adults as ages 18–21.

#### COVID-19 attitudes

Participants reported whether they personally knew someone infected by the coronavirus, and they were asked to rate their concern about becoming infected with COVID-19 on a scale from 1 (very worried) to 3 (not worried at all).

#### Other participant characteristics

Participants reported their race/ethnicity, gender identity, and sexual orientation in the baseline survey and their school enrollment, employment status, and living arrangement in the supplemental survey.

### Analysis

We used the chi-square test of independence to compare outcomes by age group, gender identity, sexual orientation, knowing someone infected by COVID-19, and concern about becoming infected. All analyses were conducted using Stata (StataCorp, College Station, TX), version 16.

To qualitatively analyze the open-ended question about the impact of the pandemic on their romantic relationships, we used a modified form of grounded theory in which an initial set of potential themes were identified based on the research’s key areas [36]. Two researchers reviewed the responses, created new codes based on emerging themes, and coded responses by theme. Researchers met to review the process, clarify codes, and make minor modifications to the coding to improve reliability. Responses were also classified by age, gender identity, and sexual orientation.

## Results

### Sample characteristics

Overall, 351 of 797 individuals responded to the supplemental COVID-19 survey (44% response rate), with 205 providing responses to the open-ended question about the effects of COVID-19 on their romantic relationships. Respondents to the supplemental survey were significantly younger, more likely to be female, Hispanic, have Internet access at home and on their phone, and less likely to be sexually experienced than non-respondents.

The majority of participants were female (72%), Hispanic (75%), and straight/heterosexual (83%), and the average age was 16.8 years (Table 1). Most participants (59%) were in middle or high school, 16% were in college, and 25% were no longer in school. Adolescents were significantly more likely to be in school and less likely to have a job than young adults. Most participants lived with their parents, including 95% of adolescents and 83% of young adults (*p* < 0.001). One quarter knew someone who had been infected by COVID-19, and most were very worried (17%) or somewhat worried (56%) about becoming infected themselves.

**Table 1:** Descriptive characteristics of the sample (*N* = 351)

	Total (*N* = 351)		Ages 13–17 (*n* = 223, 63.5%)		Ages 18–21 (*n* = 128, 36.5%)		*P*-value*
	n	%	n	%	n	%	
Race/ethnicity							0.130
Hispanic	260	75.1	171	78.1	89	70.1	
Non-Hispanic White	16	4.6	10	4.6	6	4.7	
Non-Hispanic Black	16	4.6	6	2.7	10	7.9	
Non-Hispanic Other	54	15.6	32	14.6	22	17.2	
Gender							0.073
Female	250	71.6	168	75.7	82	64.6	
Male	94	26.9	50	22.5	44	34.7	
Transgender	3	0.9	2	0.9	1	0.8	
Gender-queer/Non-binary	2	0.6	2	0.9	0	0.0	
Sexual orientation							0.569
Straight/heterosexual	285	82.9	179	81.7	106	84.8	
LGBQ	46	13.4	30	13.7	16	12.8	
Questioning/not sure	13	3.8	10	4.6	3	2.4	
School enrollment							< 0.001
In middle/high school	206	59.0	191	86.0	15	11.8	
In college	57	16.3	9	4.1	48	37.8	
Not in school	86	24.6	22	9.9	64	50.4	
Full or part-time job	70	20.2	23	10.4	47	37.3	< 0.001
Living with parents	320	91.4	215	96.4	105	82.7	< 0.001
Knows someone infected by coronavirus	84	25.1	54	25.1	30	25.0	0.981
How worried are you that you will catch COVID-19?							0.465
Very worried	59	17.7	35	16.4	24	20.0	
Somewhat worried	188	56.3	119	55.6	69	57.5	
Not at all worried	87	26.1	60	28.0	27	22.5	

* *p*-value based on chi-square test comparing adolescents (ages 13–17) and young adults (ages 18–21)

### Physical distancing from intimate partners during the COVID-19 pandemic

More than a third (37%) were dating or in a romantic relationship during the COVID-19 pandemic, including 33% of adolescents and 43% of young adults (*p* = 0.080) (Table 2). More than a quarter (28%) of all youth and 75% of those in a relationship spent time in person with their partner in the last 3 months. Young adults were more likely to spend time with a partner than adolescents (38% vs. 23%, *p* = 0.003). Eleven participants moved in with their partner in the last 3 months as a result of the pandemic, including eight young adults and three adolescents (not shown).

**Table 2:** Adolescent and young adults’ romantic relationships, physical distancing, and sexual activity during the COVID-19 pandemic

	Total (*N* = 351)		Ages 13–17 (*n* = 223)		Ages 18–21 (*n* = 128)		*P*-value*
	n	%	n	%	n	%	
**Romantic relationships and physical distancing**							
Had an intimate partner in the past 3 months	120	36.5	70	33.0	50	42.7	0.080
Spent time in person with an intimate partner in the past 3 months	92	27.9	48	22.5	44	37.6	0.003
**Sexual activity**							
Ever had oral, vaginal, or anal sex	105	31.9	54	25.5	51	43.6	0.001
Had oral, vaginal, or anal sex in the past 3 months	74	22.3	37	17.3	37	31.4	0.003

* *p*-value based on chi-square test comparing adolescents (ages 13–17) and young adults (ages 18–21)

**Fig. 1:**
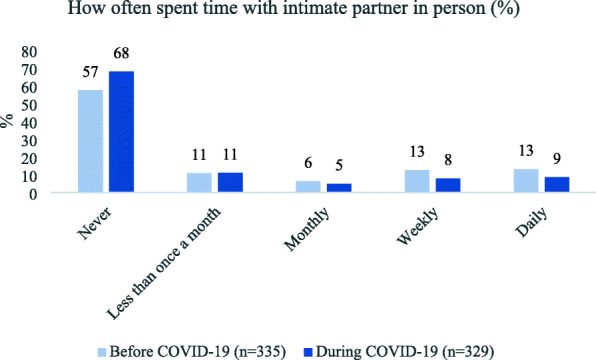
Frequency of spending time with an intimate partner, before and during COVID-19 restrictions

Youth spent time with a partner in person less often during the pandemic than before (Fig. 1). The percentage who saw a partner weekly declined from 13 to 8%, while those who saw a partner daily declined from 13 to 9%. A greater percentage of young adults than of adolescents spent time with a partner daily before COVID-19 (22% vs. 8%, *p* = 0.004) and during the pandemic (18% vs. 3%, *p* < 0.001).

In response to the open-ended question, several participants mentioned they had not dated anyone since the start of shelter-in-place restrictions. One youth stated, “I was finally starting to get around to dating, but COVID-19 really put a stop on that” (female, lesbian, gay, bisexual, or queer [LGBQ], age 19). Among those who were currently in a relationship, the majority said they had reduced or stopped spending time together in person. One responded, “I haven’t seen my boyfriend and when I have I had to wear a mask” (female, straight, age 16).

Several participants also commented that being physically apart from their partners negatively affected their relationships, fueling more arguments. One explained, “It’s made things more difficult; we’ve become more distant and fought way more than usual” (female, straight, age 18). However, others mentioned that they increased the amount of time communicating in other ways such as via FaceTime, texts, and calls. One youth explained, “It makes it hard for me and my boyfriend but we text every day and try to make the most of it” (transgender, pansexual, age 16). Another participant stated, “At first it ruined it due to me being on a stay at home order and couldn’t see them, but later on it helped the both of us a lot in ways that we maybe needed that time apart and even got to learn to communicate better” (female, straight, age 19).

#### Reasons for physical distancing from partners

Among the participants who were dating or in a romantic or sexual relationship, one third (34%) did not spend time in person with their partner because their parents would not let them go out due to COVID-19 (34%) (Table 3). In addition, 17% said that their partner’s parents would not let them go out because of the pandemic. Twelve percent felt worried about becoming infected, and 7% cited their partner’s concerns about becoming infected. One-fifth (21%) had not seen their partner due to lack of transportation.

**Table 3:** Among participants in a sexual or romantic relationship in the past 3 months, reasons for not spending time in person with their partner

	Total (*n* = 118)		Ages 13–17 (*n* = 69)		Ages 18–21 (*n* = 49)		*P*-value*
	n	%	n	%	n	%	
My parents wouldn’t let me go out because of the coronavirus	40	33.9	30	43.5	10	20.4	0.009
My date/partner’s parents wouldn’t let them go out because of the coronavirus	20	17.0	16	23.2	4	8.2	0.032
I was worried about getting the coronavirus	14	11.9	8	11.6	6	12.2	0.914
My date/partner was worried about getting the coronavirus	8	6.8	7	10.1	1	2.0	0.084
Lack of transportation	25	21.2	13	18.8	12	24.5	0.459
We broke up/stopped dating	10	8.5	7	10.1	3	6.1	0.439
I didn’t have time	17	14.4	8	11.6	9	18.4	0.302

* *p*-value based on chi-square test comparing adolescents (ages 13–17) and young adults (ages 18–21)

Compared to young adults, adolescents were more likely to physically distance from their partners due to rules of their parents (44% vs. 20%, *p* = 0.098) or their partner’s parents (23% vs. 8%, *p* = 0.032). As expected, youth who were worried about getting the coronavirus were more likely to physically distance from partners due to concerns about infection. Among young adults, the most common reason for physically distancing from partners was lack of transportation (25%).

Most youth agreed (47%) or strongly agreed (12%) that “it is okay for intimate partners to spend time together in person during shelter-in-place if they are only dating one person,” while 29% disagreed and 13% strongly disagreed with this statement (not shown). Attitudes towards physically distancing from partners were similar across age groups.

Reasons for physical distancing in the open-ended responses also included parents’ restrictions and concern about becoming infected or infecting others. One youth stated, “At first, we didn’t see each other for over a month just for the fact to be safe because we both work around a lot of people” (female, straight, age 20). Another said, “I was afraid of seeing my partner because I was still visiting my sibling” (female, straight, age 19). A couple participants mentioned that school closures meant they could not see their partners on campus. As one explained, “School isn’t happening and I don’t get to see my boyfriend every day” (female, straight, age 17).

### Sexual activity during the COVID-19 pandemic

In the supplemental survey, a third of youth (32%) indicated that they had ever had oral, vaginal, or anal sex, and 22% had sex in the past 3 months during the COVID-19 pandemic. When we compared reports of sexual activity in the past 3 months from the baseline and supplemental COVID-19 surveys, 69% were not sexually active before or during the pandemic, 14% of participants were sexually active in both periods, 9% were sexually active before COVID-19 only, and 8% were during the pandemic only.

**Fig. 2:**
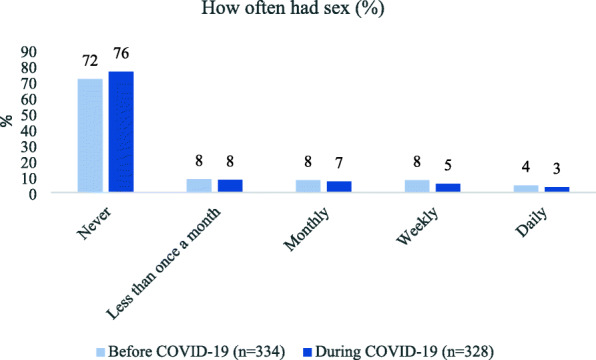
Frequency of having sex, before and during COVID-19 restrictions

Compared to adolescents, young adults were significantly more likely to have ever had sex (44% vs. 26%, *p* = 0.001) and sex in the past 3 months (31% vs. 17%, *p* = 0.003). Compared to youth who identified as straight/heterosexual, LGBQ youth were more likely to ever have had sex (53% vs. 28%, *p* < 0.001) and sex in the past 3 months (34% vs. 20%, *p* = 0.024). Participants had sex slightly less often during the pandemic. The percentage of participants who had sex monthly declined from 8 to 7%, weekly declined from 8 to 5%, and daily declined from 4 to 3% (Fig. 2).

In the open-ended responses, few focused on sexual activity, though one did mention that it affected her “relationship by not allowing contact and intimacy” (female, LGBQ, age 15). Another explained, “I haven’t had a extremely sexual relationship because I have strict parents. I seen my partner once ever[y] week so there wasn’t a huge impact” (female, LGBQ, age 17). One respondent noted, “some people want to wait until the coronavirus is over before they hangout so it blocks the act from happening” (female, straight, age 18).

### Romantic or sexual interactions online

Overall, youth were not pursuing romantic or sexual relationships online during the pandemic. Three-quarters of youth (75%) indicated that they are not involved in sexting (Table 4). Eight percent of participants reported that they spend more time sexting, 6% spend less time, and 11% spend the same amount of time sexting due to COVID-19 and shelter-in-place restrictions. Compared to young adults, adolescents were more likely to report that they do not sext (81% vs. 62%, *p* = 0.002) and less likely to report more time sexting due to COVID-19 and shelter-in-place (11% vs. 6%, *p* = 0.002).

**Table 4:** Self-reported change in the amount of time on online romantic or sexual interactions due to COVID-19 and shelter-in-place

	Total (N = 351)		Ages 13–17 (*n* = 223)		Ages 18–21 (*n* = 128)		*P*-value*
	n	%	n	%	n	%	
Sexting							0.002
More time	25	7.7	12	5.7	13	11.2	
About the same	36	11.0	15	7.1	21	18.1	
Less time	21	6.4	12	5.7	9	7.8	
I do not do this/does not apply	245	74.9	172	81.5	73	62.9	
Online dating/hook up sites							< 0.001
More time	15	4.6	4	1.9	11	9.3	
About the same	14	4.3	5	2.4	9	7.6	
Less time	14	4.3	5	2.4	9	7.6	
I do not do this/does not apply	285	86.9	196	93.3	89	75.4	
Watching porn							0.061
More time	35	10.7	21	10.1	14	12.0	
About the same	35	10.7	18	8.6	17	14.5	
Less time	25	7.7	12	5.7	13	11.1	
I do not do this/does not apply	231	70.9	158	75.6	73	62.4	

* *p*-value based on chi-square test comparing adolescents (ages 13–17) and young adults (ages 18–21),

Most youth (87%) were not using online dating/hook up sites before or during the COVID-19 pandemic. Five percent said they spend more time online dating, 4% about the same amount of time, and 4% less time online dating due to the pandemic. Online dating was more common among young adults than adolescents (25% vs. 7%, *p* < 0.001) and among male than female participants (25% vs. 9%, *p* < 0.001).

The majority of youth (71%) indicated that they do not watch porn. One in ten participants (11%) spent more time watching porn due to COVID-19, 8% spent less time, and 11% spent the same amount of time. There were no significant age differences in time spent watching porn due to COVID-19, although watching porn was almost twice as common among males than females (46% vs. 23%, *p* < 0.001) and among straight/heterosexual than LGBQ participants (49% vs. 25%, *p* < 0.001).

Only two participants mentioned online dating and none discussed porn in the open-ended responses. One stated, “I feel like nobody has anything better to do at home [so] guys have been more bold recently and I’ve been getting way more messages on social media than I usually would” (female, straight, age 16). Another explained, “I wanted to try tinder or the other dating app so that I could go on dates but the dates would have to be online now” (female, LGBQ, age 19).

## Discussion

This study examined how social distancing measures to reduce the spread of COVID-19 early in the pandemic affected youth’s involvement in romantic and sexual relationships. We found that youth who were in romantic relationships spent less time in person with their partners during the COVID-19 pandemic than before, and one quarter spent no time in person with their partner during the first 3 months of social distancing. Romantic relationships are central to adolescent development [8, 9] and prolonged separation may have lasting impacts on youth. Studies have shown elevated levels of depression and anxiety among youth during the pandemic [37–39], suggesting that many will need ongoing mental health support. At the same time, our qualitative findings suggest that being apart led to improved communication in some youth relationships.

We did not find evidence that social distancing measures resulted in less partnered sex or increased online dating among youth, as others have posited [10]. In fact, most youth surveyed were not sexually active, participating in online dating, or sexting, before or during the pandemic. Previous studies in adult populations found significant declines in sexual activity during the pandemic, although adults did not completely stop engaging in sexual activity [11–14]. Further research is needed to understand the impact of the end of pandemic restrictions on adolescents’ romantic relationships and sexual behaviors, as researchers have posited there may be a “catch-up period” in which sexual behavior increases [10].

Experiences in romantic and sexual relationships during the COVID-19 pandemic varied between adolescents and young adults, which suggests the importance of studying the impact of the pandemic and other emergencies through a developmental perspective. Young adults were more likely to spend time with their partner and to be sexually active before and during the pandemic than adolescents. These results are consistent with the increase in autonomy that typically occurs in the transition to adulthood, along with other changes such as moving away from family and entering the labor market [40].

Study findings reinforce the importance of ensuring that young people have continued access to sexual and reproductive health care during the ongoing pandemic. While some youth have continued to be sexually active, due to shelter-in-place restrictions and limitations to in-person clinical services, contraception and other services have become more difficult to access [41–43]. Many healthcare providers have quickly adapted to offer services through telemedicine during the pandemic, although more research is needed to improve the reach and appropriateness of telemedicine among adolescents and young adults [44, 45]. Researchers have posited that sexually transmitted infections may have declined due to increased isolation and less casual sex during the pandemic, although others are concerned that the lack of testing and treatment may lead to an increase in transmission [46, 47]. Health providers need to heighten efforts to provide testing to adolescents and consider home-based or self-testing. The COVID-19 pandemic has highlighted potential strategies to reduce barriers to access including telemedicine visits, home-based testing, and online prescriptions that could continue as clinics resume in-person visits [48].

With fewer resources and the increased demands on schools due to the pandemic and transition to remote instruction, sexual health education may be of lower priority. However, adolescents continue to need accurate and age-appropriate sexual health information from a trusted source. Schools and community-based organizations may need support to proceed with sexual health education in an online or digital format. These alternative formats may be applicable in the future when other health concerns or emergencies, such as wildfires, cause school closures.

This study has limitations. First, the use of retrospective and single-item measures increased the risk of measurement error. Second, the study used a sample of youth participating in an ongoing study in Fresno County, California, and the sample is not representative of youth in the county or California. Respondents to the supplemental survey were significantly younger, more likely to be female, Hispanic, have Internet access at home and on their phone, and less likely to be sexually experienced than non-respondents. The level of sexual activity during the COVID-19 pandemic may be higher in the general population of youth. Third, because of the limited sample size, the results of comparisons by gender and sexual orientation must be interpreted cautiously, and we were unable to examine other important characteristics, such as housing status. A higher response rate may have been achieved with additional time and efforts at follow up and/or a gift certificate of greater value. However, the results highlight the experiences of a predominately Hispanic population who are often underrepresented in research and media depictions of COVID-19.

As the COVID-19 pandemic evolves over time, so will its impact on youth and their relationships. It will be important to continue to study the short and long-term impacts of the pandemic on youth relationships and their sexual health. Future research should assess if the COVID-19 pandemic resulted in a lasting shift in relationship development among adolescents and young adults, including changes in their approach to casual sex, communication styles, and use of online dating. In addition, future research should examine not only the negative impacts of the pandemic, but also young people’s resiliency and post-pandemic growth in their relationships and lives.

## Conclusions

This is one of the first studies to examine the immediate impacts of social distancing requirements on young people’s romantic and sexual relationships, asking about their experiences during the first 3 months of the shelter-in-place restrictions. We found that adolescents and young adults have continued to engage in sexual and romantic relationships during the pandemic, although many reported physical distancing from their partners. Most youth were not sexually active before or during the pandemic, yet nearly a quarter had sex during this period. The results demonstrate that youth continue to need access to sexual health education and services during emergencies such as the COVID-19 pandemic.

## Data Availability

The datasets analysed during the current study are available from the corresponding author on reasonable request.
